# Special Issue “Novel Diagnostic Technologies for SARS-CoV-2 and Other Emerging Viruses”

**DOI:** 10.3390/v16081252

**Published:** 2024-08-05

**Authors:** Yohei Kurosaki, Danyelly Bruneska Gondim Martins, José Luiz Lima Filho

**Affiliations:** 1 National Research Centre for the Control and Prevention of Infectious Diseases (CCPID), Nagasaki University, Nagasaki 852-8523, Japan; 2 Keizo Asami Institute (iLIKA), Federal University of Pernambuco (UFPA), Recife 50670-901, Brazil; bruneska@prospecmol.org (D.B.G.M.); joseluiz60@gmail.com (J.L.L.F.)

In the last decade, extensive and borderless viral disease outbreaks have been caused by Ebola, Zika, and SARS-CoV-2. A new global pandemic will undeniably occur shortly. In the case of COVID-19, many individuals have been tested to confirm viral infection, and genetic variants of the virus have emerged. Several variants have caused severe public health concerns, as they might have enhanced the potential for human-to-human transmission. In molecular diagnostic laboratories, RT-PCR has been the gold standard for detecting emerging and re-emerging viruses, including SARS-CoV-2; however, RT-PCR testing is generally time-consuming and requires individuals with mature experimental skills in molecular biology. To address these obstacles, automatic virus detection systems using clinical specimens and rapid virus detection with isothermal nucleic acid amplification technologies have been developed and were available even in clinical hospitals during the COVID-19 pandemic [1,2,3].

Furthermore, recent advances in next-generation sequencing (NGS) techniques and global sequence-sharing networks have rapidly described the emergence of concern variants and global pathways of these variant transmissions [4,5]. In this Special Issue, we focused on novel and effective technologies for detecting viruses or biomarkers that indicate viral infection using nucleic acid amplification, antigen detection, NGS, and other germination techniques that could be applied to detect emerging viruses. We have featured four original articles and two review articles in this Special Issue.

Since the pandemic started in 2019, a sensitive and specific SARS-CoV-2 detection biosensor has been required, especially for patients with severe acute respiratory syndrome. Several detection methods have been applied to identify viral particles in biological samples during pandemics. Bojórquez-Velázquez et al. demonstrated the importance of applying new technologies for virus detection in food and environmental samples, particularly for SARS-CoV-2 [6]. Using mass spectrometry, they detected the virus more quickly and found new variants because they could directly determine the amino acid sequences of the viral proteins in the specimens. Proteomics and mass spectrometry are expected to play important roles in the epidemiological control of COVID-19 and other diseases.

Mugnier et al. developed an easy-to-use web-based application (EPISEQ SARS-CoV-2) to analyze SARS-CoV-2 NGS data generated on common sequencing platforms using a variety of commercially available reagents, helping laboratories with limited bioinformatic capacity [7]. A comparison of several sequencing approaches using EPISEQ SARS-CoV-2 revealed 100% concordance in the clade and lineage classifications. This study also revealed reagent-related sequencing issues that could impact SARS-CoV-2 mutation reporting. This application made it easy to access and translate the raw NGS data.

Many patients who recovered from COVID-19 continue to experience various symptoms long past the time of their initial recovery, referred to as Post-Acute Sequelae of SARS-CoV-2 infection (PASC) or “long COVID”. Happel et al. demonstrated the National Institutes of Health initiative for novel exosome-based technologies for detecting SARS-CoV-2. The present study could detect SARS-CoV-2 RNA and protein combinations, exosomal RNA and proteins, and host antibodies by developing SARS-CoV-2 multiparametric assays [8]. Additionally, it can provide clinically relevant information, such as the presence of broadly neutralizing antibodies or prognostic biomarkers for severe COVID-19 or PASC. 

Due to its high sensitivity and specificity, Zheng et al. developed a quantum dot technology that is a very promising way to achieve this [9]. It is a nanometer-scale fluorescent biosensor system consisting of CdSe-ZnS quantum dots (QDs) coupled with the highly sensitive B-cell epitopes of SARS-CoV-2, which can remarkably identify the corresponding antibody with a detection limit of 100 pM. Intriguingly, the authors found that the fluorescence quenching of QDs was stimulated more obviously when coupled with peptides than with the corresponding proteins, indicating that the biosensor provides a novel real-time, quantitative, and high-throughput method for clinical diagnosis and home-use tests.

Infectious virus and pseudotyped virus entry assays detect functionally active antibodies but are limited by biosafety and standardization issues. Abdul et al. reported that not all antibodies against SARS-CoV-2 inhibited viral entry and infection [10]. Neutralizing antibodies are more likely to reflect natural immunity; however, specific tests have investigated protein–protein interactions rather than fusion events. Based on this, they developed a SARS-CoV-2 Spike protein-and cellular receptor angiotensin-converting enzyme 2 (ACE2)-dependent fusion assay based on split luciferase. This new method consists of rapid execution (24 h) in multiwell plates, compared to the plaque reduction neutralization test and pseudotyped viruses, and can be performed in a standard biosafety environment. It provides robust reproducibility and standardization and is adapted for automation and large-scale studies.

Understanding the integration sites between the B-cell lymphoma lines BLSC-KU1 and BLSC-KU17 and applying the viral DNA capture high-throughput sequencing system are fundamental to avoid bovine leukemia. Yamanaka et al. showed for the first time the development of a novel proviral DNA-capture sequencing method that investigated bovine leukemia virus (BLV) proviral integration in two B-cell lymphoma lines, BLSC-KU1 and BLSC-KU17, derived from BLV-infected cattle with enzootic bovine leukosis [11]. This is an innovative method for screening BLV-infected cattle at an earlier stage than those that have already developed lymphomas.

In this Special Issue, we provide information on novel diagnostic methods targeting different phases of infection (acute and convalescent) and different approaches (the virus itself and host immunological responses). In response to the next pandemic, cost-effective, labor-saving, and high-throughput molecular diagnostic technologies must be continuously developed for detecting viruses and their genetic mutations. Furthermore, the surveillance of zoonotic viruses is necessary to prepare for the subsequent spillover of known or unknown pathogenic viruses from nature to human communities.

## Data Availability

No new data were created or analyzed in this study. Data sharing is not applicable to this article.
